# Development of a Pilot Introductory Advanced Cardiovascular Resuscitation Course for Senior Medical Students in Switzerland: Student-Driven Implementation Study

**DOI:** 10.2196/46075

**Published:** 2023-06-27

**Authors:** Tara Herren, Loris Fivaz, Eva Dufeil, Eric Golay, Ely Braun, Emilie Straub, Philippe Nidegger, Olivier Grosgurin, Birgit Andrea Gartner, Mélanie Suppan, Laurent Suppan

**Affiliations:** 1 Division of Emergency Medicine Department of Anesthesiology, Clinical Pharmacology, Intensive Care and Emergency Medicine University of Geneva Hospitals and Faculty of Medicine Geneva Switzerland; 2 Division of Anesthesiology Department of Anesthesiology, Clinical Pharmacology, Intensive Care and Emergency Medicine University of Geneva Hospitals and Faculty of Medicine Geneva Switzerland

**Keywords:** advanced cardiovascular life support, undergraduate medical education, cardiopulmonary resuscitation, CPR, medical education, resuscitation, web-based questionnaire, collaborative design, implementation, medical course, curriculum, student, life support, training, cardiac arrest, medical student

## Abstract

**Background:**

Cardiac arrest is the most time-critical emergency medical students and junior physicians may face in their personal or professional life. However, many studies have shown that most of them lack the necessary knowledge and skills to efficiently perform resuscitation. This could be related to the fact that advanced cardiovascular resuscitation courses are not always part of the undergraduate medical curriculum.

**Objective:**

The aim of this study was to describe the development, pilot implementation, and assessment of an advanced cardiovascular resuscitation course designed to enable senior medical students to manage the initial resuscitation phase in case of cardiac arrest.

**Methods:**

An introductory advanced cardiovascular resuscitation course was developed on the initiative of fifth-year medical students, in collaboration with the prehospital emergency medical service team of the Geneva University Hospitals. The 60 slots available to the 157 members of the fifth-year promotion of the University of Geneva Faculty of Medicine were filled in less than 8 hours. This unexpected success prompted the creation of a first questionnaire, which was sent to all fifth-year students to determine the overall proportion of students interested in attending an advanced cardiovascular resuscitation course. This questionnaire was also used to assess basic life support education and experience among course participants. A postcourse questionnaire was used to gather feedback regarding the course and to assess student confidence regarding the resuscitation skills they had been taught.

**Results:**

Out of 157 fifth-year medical students, 73 (46%) completed the first questionnaire. Most thought that the current curriculum did not provide them with enough knowledge and skills regarding resuscitation and 85% (62/73) wished to attend an introductory advanced cardiovascular resuscitation course. All the participants who would have wanted to follow the full Advanced Cardiovascular Life Support course before graduating were set back by its cost (10/10, 100%). Of the 60 students who had registered for the training sessions, 56 (93%) actually attended. The postcourse questionnaire was completed by 42 (87%) students (out of 48 who had registered on the platform). They unanimously answered that an advanced cardiovascular resuscitation course should be part of the standard curriculum.

**Conclusions:**

This study demonstrates the interest of senior medical students in an advanced cardiovascular resuscitation course and their willingness to see such a course integrated as a part of their regular curriculum.

## Introduction

### Background

Senior medical students about to graduate are sometimes expected to have skills close to those of certified physicians even though their training is still ongoing [1]. During the COVID-19 pandemic, these expectations were particularly emphasized, and many senior undergraduate students were given responsibilities akin to those of residents [2]. Regardless of the context of this recent crisis, any medical student can be exposed to critical emergencies whether inside or outside the hospital [1,3,4]. Indeed, they are expected to adequately manage resuscitations even though they have not yet graduated [5]. Moreover, in Switzerland as in many other countries, newly graduated physicians spend their first year of residency in peripheral hospitals where senior practitioners are not constantly present. These physicians should therefore be able to manage the first 10 minutes of resuscitation without external help. However, many studies show that senior medical students who are about to graduate are not proficient in resuscitation skills despite significant improvements throughout their curriculum [5-10]. Furthermore, most senior medical students feel unconfident putting these skills into action [1,11]. This feeling could be explained by the paucity of refresher sessions during their undergraduate curriculum and by the impression that their resuscitation training is not comprehensive or advanced enough [12].

### Local Setting

During their 6-year curriculum, medical students at the University of Geneva Faculty of Medicine (UGFM), Switzerland, follow 4 basic life support (BLS) training sessions. Even though the last of these sessions, which takes place during their fifth year, confronts them with more difficult cases requiring a better understanding of the pathophysiology of cardiac arrest (CA), they are not expected to practice skills beyond standard BLS procedures.

In 2021, a small group of fifth-year medical students was interested in taking an Advanced Cardiovascular Life Support (ACLS) course since they were concerned about their resuscitation skills. Since this course is both expensive and time-consuming, they contacted the prehospital emergency medical service (Mobile Emergency and Resuscitation Service [SMUR for Service Mobile d’Urgence et de Réanimation, in French]) of the Geneva University Hospitals to determine if an introductory advanced cardiovascular resuscitation course could be organized free of charge. A total of 60 training slots were provided to the 157 students of the fifth-year promotion, all of which were filled in less than 8 hours. This unexpected enthusiasm prompted the elaboration of a questionnaire to explore fifth-year medical students’ support of the inclusion of an introductory advanced cardiovascular resuscitation course during the undergraduate emergency training program as part of the standard curriculum.

### Objectives

The goal of this study was to describe the development, pilot implementation, and assessment of an introductory advanced cardiovascular resuscitation course designed to enable senior medical students to manage the first 10 minutes of resuscitation in case of CA.

## Methods

### Ethics Approval

In accordance with the Swiss federal law on human research, the regional ethics committee (Commission Cantonale d'Ethique de la Recherche sur l'être humain—CCER—Geneva, Switzerland) issued a “declaration of no objection” (Req-2021-00628) regarding this study [13].

### Study Design

This was a retrospective analysis of data collected prospectively through fully automated web-based questionnaires. Participants were informed that anonymized data would be collected and presented to the UGFM committee for undergraduate education. The students were also informed by email that a publication was considered and had the opportunity to ask questions and express their potential opposition. Methods and results are reported according to the CHERRIES (Checklist for Reporting Results of Internet E-Surveys) guidelines [14].

### Enrollment and Precourse Questionnaire

Information about the course was dispatched to all fifth-year medical students by email on February 18, 2021, and was simultaneously posted on the social network used for the promotion. The fifth-year UGFM promotion included 157 students representing our convenience sample. Students could register for the course by responding to the invitation email on a first come, first served basis. No financial incentive was given to promote participation, and there were no exclusion criteria.

A web-based platform was created using the Joomla 3.9 content management system (Open Source Matters) to host the web-based questionnaires, which were created using the Community Surveys 5.5 component (Shondalai). The platform and questionnaires were thoroughly tested by 4 of the authors. The first questionnaire was sent to the participants along with practical information regarding the training sessions on April 1, 2021, and a reminder was sent 3 days later. This questionnaire was divided into 2 sections (Table 1). The aim of the first section was to determine the potential number of students interested in such a training and was intended for the all fifth-year UGFM students, regardless of their participation in or interest for the course. Registered participants accessed the second part, which was specifically designed to assess their comfort with the BLS skills they had acquired through the standard training curriculum and their desire to follow an official ACLS course prior to registering to the introductory advanced cardiovascular resuscitation course. After completing this part of the questionnaire, participants were prompted to create an account on the web-based platform.

To make sure that participants would reap the highest possible benefit from the course and to facilitate the flow of the practice sessions, they were asked to read a summary of the ACLS guidelines [15] before attending the course.

**Table 1:** Precourse questionnaire.

Survey page, field, and questions				Type of question
**Page 1**				
	**Demographics**			
		Age		Open^a^
		Gender		MCQ^b^
	**Knowledge and interest about ACLS^c^**			
		Ever heard of ACLS		Yes or no
		Benefits expected from attending an ACLS course		MAQ^d^
	**Regarding the class organized this year**			
		**Registration**		Yes or no
			If yes: go to questions on page 2	
			If no: wished they could have participated	Yes or no
**Page 2**				
	**BLS^e^ awareness**			
		Number of BLS-AED^f^ courses attended		MCQ
		Ability to use the already acquired BLS knowledge		1-5 Likert scale
		Ever performed chest compression in a real situation		Yes or no
	**Interest in taking an ACLS class before graduation**			
		Wish to obtain an ACLS certification		Yes or no
		Impeding factors		MAQ
		Advanced resuscitation course as part of the curriculum		1-5 Likert scale
	**Interest in emergency medicine**			
		Willingness to specialize in emergency medicine		MCQ

^a^A Regex (regular expression) validation rule was used to avoid invalid entries.

^b^MCQ: multiple-choice question (only 1 answer accepted).

^c^ACLS: advanced cardiovascular life support.

^d^MAQ: multiple-answer question (more than 1 answer accepted).

^e^BLS: basic life support.

^f^AED: automatic external defibrillator.

### Design and Sequence of the Course

There were 5 training sessions between April 10 and June 12, 2021, and students were divided into groups accordingly. The course was designed by the SMUR team in collaboration with senior medical students whose pre-existing knowledge was taken into account. Course sessions lasted 5.5 hours and were scheduled during the weekends to reduce the impact on the standard teaching curriculum. Their structure, mostly based on current ACLS guidelines [11], is detailed in Table 2.

Three main themes were covered during the simulation sessions: rhythm recognition and management, drug use and timing of drug administration, and specialized care after the return of spontaneous circulation. Nontechnical skills such as leadership and communication were also practiced during these simulations. Regarding airway management procedures, students were taught to prepare and insert an i-gel supraglottic airway device since such devices enhance oxygenation and ventilation and do not require the level of expertise needed to perform endotracheal intubation [16].

To ensure a personalized and efficient training experience, there was a ratio of 1 instructor for 4 medical students. This also helped adhere to the COVID-19 infection prevention guidelines, which were in effect at the time of this study.

All instructors were SMUR paramedics certified in BLS training and used to teach advanced resuscitation skills. In Switzerland, paramedics follow a 3-year curriculum and are able to take care autonomously of a wide range of injured or ill patients of all ages. They are allowed to insert intravenous lines, use supraglottic airway devices, and administer a wide variety of drugs including epinephrine, antiarrhythmic agents such as amiodarone, and opiates such as fentanyl [17]. Paramedics staffing SMUR units possess additional skills and have access to advanced medications such as hypnotics and neuromuscular blocking agents. These advanced paramedics always work in pairs with physicians who are either senior residents, registrars, or even senior specialists in prehospital emergency medicine [18].

An unofficial course completion certificate was given to the students at the end of the course.

**Table 2:** Contents of the introductory advanced life support training course.

Topic and method		Duration (minutes)
**Primary survey**		
	Theoretical explanation and demonstration	30
**BLS-AED^a^ refresher**		
	Workshop	45
**Airway management procedures**		
	Theoretical explanation, demonstration, and practice	30
**Team dynamics during cardiac arrest situations**		
	Demonstration	25
	Simulation and debriefing	90
**Immediate care after ROSC^b^**		
	Demonstration	30
	Simulation and debriefing	90

^a^BLS-AED: basic life support and automatic external defibrillator.

^b^ROSC: return of spontaneous circulation.

### Postcourse Questionnaire

A few hours after the end of the training session, participants received an email containing a link to the postcourse questionnaire (Table 3). The goal of this questionnaire, which was administered using the same platform, was to determine whether participants thought that this course should be integrated into the regular curriculum.

Since participants had to be registered on the platform to answer this last questionnaire, we were able to send regular reminders to enhance the participation rate.

**Table 3:** Postcourse questionnaire.

Survey page, field, and questions					Type of question
**Page 1**					
	**General opinion**				
		**Liked the course?**		Yes or no	
			If yes: why	MAQ^a^	
			If no: why	MAQ	
		**Was the course format adequate?**		Yes or no	
			If no: why	MCQ^b^	
		Suggestions for improvement		Open	
		Usefulness of the content		1-5 Likert scale	
		Met expectations		1-5 Likert scale	
	**Link with faculty program**				
		Should be integrated in the standard curriculum		1-5 Likert scale	
	**Confidence**				
		Confidence in their ability to apply the knowledge learnt		1-5 Likert scale	

^a^MAQ: multiple-answer question (more than 1 answer accepted).

^b^MCQ: multiple-choice question (only 1 answer accepted).

### Outcomes

The primary outcome was the proportion of fifth-year students wishing to follow an introductory advanced cardiovascular resuscitation course, regardless of whether they had been able to attend one of the sessions organized for their promotion. Secondary outcomes were the assessment of the motivations and impeding factors to follow such a course. Comments about the usefulness of the pilot course and potential modification, their confidence regarding the skills they learned, and their opinion on its integration into the standard curriculum were also analyzed.

### Data Curation and Statistical Analysis

All data were stored on an encrypted MySQL database (MariaDB 5.5.5) and extracted to a CSV file. Data curation and analysis were performed under Stata (version 16.1; StataCorp LLC). Incomplete questionnaires were not analyzed. The results are presented using descriptive statistics (n [%] and median [IQR]). Normality was assessed graphically, and between-group comparisons were carried out using the chi-square test, except for age for which the Mann-Whitney Wilcoxon test was used. A *P* value lower than .05 was considered significant.

## Results

### Precourse Questionnaire

Out of 157 fifth-year medical students, 73 completed the first questionnaire (73/157, 46%). There was no opposition from students regarding the use of their answers for publication. Participation was significantly higher (*P*<.001) in the subgroup of students who had registered to attend a training session (48/60, 80%). The characteristics of the respondents who had been able to register were not different from those who had not been able to register (Table 4).

The proportion of participants who wished to attend an introductory advanced cardiovascular resuscitation course was 85% (62/73). Their main motivation was that they thought it would help them to prepare better for residency (61/62, 98%). Most students were also interested in improving their resuscitation skills (53/62, 85%) and in increasing their knowledge (46/62, 74%). One student (1/62, 2%) reported an interest in the interprofessional aspect of this course that is, working alongside paramedics. Few of those who wished to attend thought that the current BLS-automatic external defibrillator (AED) curriculum provided them with enough knowledge and skills regarding resuscitation (2/62, 3%). This proportion was significantly higher (*P*=.001) in those who did not wish to attend such a course (5/11, 45%).

Students who had registered for a practice session had previously attended a median number of 4 (IQR 3-4) BLS-AED courses. Most felt confident or very confident in their BLS-AED abilities (35/48, 73%), but only a few of them had already performed cardiopulmonary resuscitation in an actual CA (4/48, 9%). Of the 10 (21%) participants motivated to follow the full ACLS course before graduating, all were set back by the cost of this course (10/10, 100%).

Of the 48 participants who completed the precourse questionnaire, 19 (40%) considered specializing in emergency medicine, intensive care medicine, or anesthesiology.

**Table 4:** Comparison of subgroups who answered the questionnaire.

Characteristics		Registered (n=48)		Unregistered (n=25)		*P* value
**Gender, n (%)**						.64
	Men	16 (33)	11 (44)			
	Women	31 (64)	14 (56)			
	Refused to answer	1 (2)	0 (0)			
Age (years), median (IQR)		24 (21-27)		24 (23-24)		.90
Knew what ACLS^a^ was before course proposition, n (%)		32 (67)		20 (80)		.28

^a^ACLS: advanced cardiovascular life support.

### Postcourse Questionnaire

Forty-two of the 48 students who had completed the precourse questionnaire filled the postcourse questionnaire (42/48, 87%). There was a 93% (56/60) attendance to the practice sessions.

All the participants who answered the postcourse questionnaire reported that they liked the course (42/42, 100%) and that they found its content and structure adequate. All participants thought that the course was either very useful (39/42, 93%) or useful (3/42, 7%). They linked their appreciation to the acquisition of new knowledge (40/42, 95%), to the perceived usefulness of the course (36/42, 86%), and to the opportunity of practicing resuscitation skills (39/42, 93%). Nine students wrote free comments. Four of these comments were directly linked to the importance of practicing resuscitation skills. One student commented “we tend to take for granted too easily the gestures in theory when we notice that it is not the case in practice.” Three students complimented the pedagogy of the instructors: “Very friendly instructors, and very interactive course.” One student acknowledged the interest of including leadership skills training in the course: “Very interesting leadership training.” One student answered that they would feel less helpless if confronted with a CA.

Most students answered that the content of the course had completely fulfilled their expectations (39/42, 93%). The most common suggestion for improvement was to provide an electronic learning (e-learning) module rather than text documents as references prior to the course.

After the course, the majority of participants (39/42, 93%) felt either confident (26/42, 62%) or very confident (13/42, 31%) in their ability to apply the skills they had been taught during the course.

Finally, all participants thought that such a course should be part of the regular curriculum, with 93% of them answering that it should “absolutely” be a part of it.

## Discussion

### Main Considerations

This study shows that many fifth-year medical students are highly supportive of the integration of an introductory advanced cardiovascular resuscitation course in their curriculum to feel better prepared for their first professional experience and the responsibilities it implies. The process which led to the creation of this advanced cardiovascular resuscitation course and the results of this study indicate that many students feel unprepared to manage time-critical emergencies such as CA, and ACLS courses are often offered too late during residency. Consequently, while BLS-AED refresher courses need to be held on a regular basis, advanced resuscitation skills should also be taught at least to senior medical students.

Conflicting with the results of many previous studies reporting that BLS skills are often lacking among health care students [5-7], most participants answered that they felt comfortable using such skills. This is of particular importance since the lack of confidence is often reported as a reason for inaction, especially in CA [19-21]. Promoting the confidence of health care students in their abilities to adequately provide resuscitation maneuvers is of paramount importance for several reasons. First, the expectations of the population toward medical students increase as they progress through their studies, and their resuscitation skills should therefore be developed enough to enable them to respond adequately in case of out-of-hospital cardiac arrest (OHCA). Second, the proportion of OHCA victims receiving BLS can and should be improved. In Geneva, Switzerland, the proportion of OHCA victims receiving BLS was lower than 40% between 2009 and 2012 [22]. Off-duty medical students could help improve this proportion, either on their own or as part of a first-responder network [21].

The disappointing results regarding resuscitation skills reported by many studies are probably linked to skill and information retention. Indeed, it has been shown that BLS-AED skills and knowledge decrease significantly within months after the last training session [23,24]. The same issue affects advanced life support skills among anesthetists [25]. The 2021 European Resuscitation Council Guidelines for education do not rule on the optimal frequency and method to prevent skill decay, which varies greatly according to the population studied [26].

Since the undergraduate medical curriculum is already very dense and demanding, there is significant tension between the importance of teaching more advanced resuscitation skills to senior medical students and the need to continue practicing basic resuscitation maneuvers. Alternative teaching methods could therefore be considered to enhance flexibility and efficiency. In line with one of the comments recorded by a student, including an e-learning module could prove worthwhile. Indeed, interactive e-learning modules and serious games have shown many advantages compared to traditional lectures and their use has been tremendously developed during the COVID-19 pandemic [27-29]. These modules tend to decrease costs since asynchronous distance learning does not require the presence of an instructor or even the availability of a classroom. However, despite the aforementioned advantages and their ability to significantly enhance knowledge acquisition [30], e-learning modules also present incontrovertible limitations since skill acquisition can hardly be achieved through theoretical interventions [31]. Blended courses, which ally the best of both worlds (e-learning and hands-on practice sessions), could therefore represent an effective solution [32,33]. This is even more important in the context of an advanced cardiovascular resuscitation course, which must include elements linked to nontechnical skills such as leadership, decision-making, and team working [34]. Advanced simulations have proven to be particularly effective in helping to develop such skills while honing technical ones [35].

### Limitations

A selection bias cannot be ruled out since the questionnaires were mostly completed by students who had already registered as participants for this course and were therefore probably more interested than some of their colleagues in this particular domain. Therefore, the postcourse confidence may be overestimated, and the lack of a similar question in the precourse questionnaire prevented the assessment of the participants’ confidence. In addition, the design of the first questionnaire, the second part of which could not be filled by the students who had not been able to register for the course may have prevented the acquisition of potentially useful data. Moreover, even though there is considerable overlap between the pregraduate medical curriculum of many universities, we must acknowledge that our convenience sample only consisted of UGFM medical students and that our results might not apply to other universities. Finally, the methods used in this study were hardly ideal. Indeed, given the unforeseen enthusiasm of senior medical students for an advanced cardiovascular resuscitation course, this was not a preplanned study, and the timing of some interventions was far from perfect (1 practice session was held during the end-of-year examinations, while another took place during the holidays). This last limitation might have dampened the participation rate.

### Perspectives

Following our initial results, the UGFM committee for undergraduate education decided to integrate a mandatory blended learning advanced resuscitation course, including an interactive e-learning module, into the pregraduate medical curriculum. The uptake of this course should now be assessed, and its potential shortcomings addressed before an assessment of its actual impact on the knowledge, skills, and confidence of senior medical students can be carried out. Assessing the impact of such a course on confidence through a thorough validated questionnaire would be most relevant according to the theory of planned behavior [36,37]. Finally, particular attention should be paid to the development of nontechnical skills (leadership, task management, decision-making, team working, and situational awareness), which deserve to be carefully assessed and improved.

### Conclusions

This study demonstrates the interest of senior medical students in an advanced cardiovascular resuscitation course and their willingness to see such a course integrated as part of their regular curriculum. Regardless of the course format, enabling senior medical students to acquire advanced life support knowledge and skills should help them manage more efficiently the time-critical emergencies they will encounter either prior to graduation or during their first years of residency.

## Abbreviations

ACLS: advanced cardiovascular life support
AED: automatic external defibrillator
BLS: basic life support
CA: cardiac arrest
CHERRIES: Checklist for Reporting Results of Internet e-Surveys
e-learning: electronic learning
OHCA: out-of-hospital cardiac arrest
SMUR: Service Mobile d’Urgence et de Réanimation
UGFM: University of Geneva Faculty of Medicine

## Data Availability

The data sets used and analyzed during this study are available from the corresponding author on reasonable request.
